# Development of a Model-Informed Dosing Tool to Optimise Initial Antibiotic Dosing—A Translational Example for Intensive Care Units

**DOI:** 10.3390/pharmaceutics13122128

**Published:** 2021-12-10

**Authors:** Ferdinand Anton Weinelt, Miriam Songa Stegemann, Anja Theloe, Frieder Pfäfflin, Stephan Achterberg, Lisa Schmitt, Wilhelm Huisinga, Robin Michelet, Stefanie Hennig, Charlotte Kloft

**Affiliations:** 1 Department of Clinical Pharmacy and Biochemistry, Institute of Pharmacy, Freie Universitaet Berlin, 12169 Berlin, Germany; ferdinand.weinelt@fu-berlin.de (F.A.W.); lisa.ehmann@fu-berlin.de (L.S.); robin.michelet@fu-berlin.de (R.M.); stefanie.hennig@certara.com (S.H.); 2 Graduate Research Training Program PharMetrX, 12169 Berlin, Germany; 3 Department of Infectious Diseases and Respiratory Medicine, Charité-Universitaetsmedizin Berlin, Corporate Member of Freie Universitaet Berlin, Humboldt-Universitaet zu Berlin, Berlin Institute of Health, 10117 Berlin, Germany; miriam.stegemann@charite.de (M.S.S.); frieder.pfaefflin@charite.de (F.P.); stephan.achterberg@charite.de (S.A.); 4 Antimicrobial Stewardship, Charité-Universitaetsmedizin Berlin, Corporate Member of Freie Universitaet Berlin, Humboldt-Universitaet zu Berlin, Berlin Institute of Health, 10117 Berlin, Germany; 5 Pharmacy Department, Charité-Universitaetsmedizin Berlin, Corporate Member of Freie Universitaet Berlin, Humboldt-Universitaet zu Berlin, Berlin Institute of Health, 10117 Berlin, Germany; anja.theloe@charite.de; 6 Institute of Mathematics, University of Potsdam, 14476 Potsdam, Germany; huisinga@uni-potsdam.de; 7 School of Clinical Sciences, Faculty of Health, Queensland University of Technology, Brisbane 4000, Australia; 8 Certara, Inc., Princeton, NJ 08540, USA

**Keywords:** model-informed dosing tool, intensive care unit, antibiotic therapy, antimicrobial stewardship, meropenem, pathogen susceptibility

## Abstract

The prevalence and mortality rates of severe infections are high in intensive care units (ICUs). At the same time, the high pharmacokinetic variability observed in ICU patients increases the risk of inadequate antibiotic drug exposure. Therefore, dosing tailored to specific patient characteristics has a high potential to improve outcomes in this vulnerable patient population. This study aimed to develop a tabular dosing decision tool for initial therapy of meropenem integrating hospital-specific, thus far unexploited pathogen susceptibility information. An appropriate meropenem pharmacokinetic model was selected from the literature and evaluated using clinical data. Probability of target attainment (PTA) analysis was conducted for clinically interesting dosing regimens. To inform dosing prior to pathogen identification, the local pathogen-independent mean fraction of response (LPIFR) was calculated based on the observed minimum inhibitory concentrations distribution in the hospital. A simple, tabular, model-informed dosing decision tool was developed for initial meropenem therapy. Dosing recommendations achieving PTA > 90% or LPIFR > 90% for patients with different creatinine clearances were integrated. Based on the experiences during the development process, a generalised workflow for the development of tabular dosing decision tools was derived. The proposed workflow can support the development of model-informed dosing tools for initial therapy of various drugs and hospital-specific conditions.

## 1. Introduction

Rational antibacterial therapy requires more than the appropriate choice of the antibiotic drug. Equally important are dosing regimens leading to an effective drug exposure linked to improved clinical success [1,2]. In intensive care unit (ICU) patients, the selection of an appropriate dosing regimen for an individual patient is challenging. The broad range of pathophysiological changes leads to high pharmacokinetic (PK) variability, which results in substantial differences in drug exposures between patients receiving the same dosing regimen [3,4,5,6,7]. To address these challenges, drug concentration measurements in combination with model-informed Bayesian dosing software have been suggested to monitor and, if needed, adjust dosing in this patient [8,9]. Unfortunately, in many hospitals, reliable, timely, and frequent concentration measurements of antibiotic drugs other than aminoglycosides are not implemented, and the use of Bayesian dosing software to inform subsequent dose adaptation is not common [10,11]. The lack of specialist expertise and structured processes, the costs for software and bioanalysis, and inconsistent global, national, and local regulations (e.g., concerning liability) impede the widespread implementation of model-informed Bayesian dosing software [12]. If software-based tools and frequent concentration measurements are not feasible, one promising alternative to individualise antibiotic therapy is tabular model-informed dosing tools or algorithms. These dosing tools can provide adequate initial dosing regimens for a wide range of patients, based on their patient characteristics and PK models of the drugs. In this context, existing PK models could be leveraged for a local patient population to circumvent the need for further PK studies.

Commonly, at the start of antibiotic therapy, neither the pathogen nor its susceptibility to the antibiotic are known. In many cases, both remain unknown during the course of antibiotic therapy [13]. Thus, patients without determined pathogen and its susceptibility are empirically treated based on the reported PK/pharmacodynamics (PD) breakpoints of the suspected pathogens [14]. The timely initiation of adequate empiric therapy is associated with decreased mortality rates, decreased length of hospitalisation, and decreased health care costs in patients with severe infections [15]. However, this strategy does usually not utilise available knowledge of a hospital regarding the susceptibility of local pathogens, and thus, it accepts the risk of unnecessary high or low and possibly toxic or ineffective antibiotic concentrations.

Meropenem is a broad-spectrum antibiotic frequently used to treat severe infections in ICU patients. It is considered to be a safe and well-tolerated antibiotic drug [16]. However, changes in meropenem PK in chronic disease patients, such as chronic kidney disease, can increase the risk for ineffective or toxic meropenem exposure [17]. The antimicrobial activity of meropenem is linked to the time period of the unbound concentration exceeding the minimum inhibitory concentration (MIC) of a pathogen. Therefore, the PK/PD index is *f*T_>MIC_ [18]. Recently, a concentration measurement program for beta-lactam antibiotics at selected ICUs of Charité-Universitaetsmedizin Berlin, a tertiary care centre with a total of >3000 in-patient beds, was initiated. Observational unpublished data from that program showed >60% of measured minimum meropenem concentrations outside the locally defined target range of one to five times MIC. Consequently, the main goal of the present study was to improve the initial meropenem therapy in ICU patients and to optimise antibiotic dosing prior to pathogen detection. Hence, this study (i) aims to develop a tabular model-informed dosing tool to optimise initial therapy of the antibiotic meropenem at Charité-Universitaetsmedizin Berlin and for this (ii) investigated how to integrate previously observed, yet unexploited local pathogen susceptibility information into dosing decisions. Ultimately, a generalised workflow for the development of tabular model-informed dosing decision tools for initial antibiotic therapy to foster their implementation at the point-of-care was developed.

## 2. Materials and Methods

### 2.1. Patient Population and Meropenem Concentration Measurements

For the development of the model-informed dosing tool, 306 routine blood samples, dosing information immediately prior to sampling, and patient-specific data of 81 ICU patients receiving meropenem therapy at two ICUs (Department of Infectious Diseases and Respiratory Medicine; Department of Surgery) at Charité-Universitaetsmedizin Berlin were collected (approval: Charité Ethics Committee, EA4/053/19). For a subset of 34 patients with 66 samples, the full dosing history was recorded.

Meropenem concentrations were determined by Labor Berlin (Labor Berlin—Charité Vivantes GmbH, Berlin). Samples were sent to the laboratory within 1 h, centrifuged, and stored at −20 °C until plasma meropenem concentrations were measured After protein precipitation with methanol, meropenem concentration was quantified using high-performance liquid chromatography (C8 reverse phase column and a 4 min step-elution gradient (0.2% HCOOH/MeOH)) coupled with tandem mass spectrometry (electrospray ionisation (ESI+) in multiple reaction monitoring). The used bioanalytical method was validated according to the protocol of the Society of Toxicological and Forensic Chemistry (GTFCh) showing good analytical performance (inaccuracy: <±5.9% relative error, imprecision: ≤6.3% coefficient of variation, calibration range: 2–30 µg/mL).

### 2.2. Pharmacokinetic Model Selection, Reduction, and Evaluation

Exclusively minimum meropenem concentrations and only 66 samples including the full dosing history were available. As a consequence, the PK data were unsuitable for PK model development. Instead, a published PK model was selected, evaluated for its appropriateness, and applied for the development of the dosing decision tool. The selection of the PK model was based on a high similarity of patient characteristics between the local study population and the model-underlying population.

To ensure the adequacy of the selected PK model for the new patient population, it was evaluated using median prediction errors and normalised prediction distribution errors (NPDEs) [19,20]. Model-predicted concentrations were obtained by 500 stochastic simulations based on the design and patient characteristics in the subset with full dosing history. To include parameter uncertainty, stochastic simulations were repeated for 1000 PK parameter sets obtained by bootstrapping of the dataset and re-estimation of the PK model. To assess possible deviations of the NPDEs from the standard normal distribution, the Wilcoxon signed-rank test (mean ≠ 0), Fisher ratio test (variance ≠ 1), and Shapiro–Wilks test (normality assumption) were used. Simulations and bootstrap analyses were performed using NONMEM 7.4.3 (ICON Development Solutions, Ellicott City, MD, USA) and PsN version 4.7.0) [21]. NPDEs were analysed using the npde package (v. 2.0) in R/Rstudio (v. 3.5.0/v. 1.1.447) [22].

### 2.3. Pharmacokinetic/Pharmacodynamic Targets

Based on current literature evidence, the PK/PD target for ICU patients receiving short-time or prolonged meropenem infusions was defined as 100%*f*T_>MIC_, while for patients receiving continuous meropenem infusions, it was defined as 100%*f*T_>4*MIC_ to prevent steady-state meropenem concentrations within the mutant selection window [23,24]. The mutant selection window refers to a range of antibiotic drug concentrations in which only the growth of the most susceptible strains of a pathogen is suppressed. As a consequence, a growth advantage is provided to already available less susceptible strains in the pathogen population. Over a longer period of time, drug concentrations within the mutant selection window increase the proportion of less susceptible pathogens and thus the risk for resistant mutations to prevail [25]. Given that 100%*f*T_>MIC_ cannot be achieved for an intravenous drug infusion on the first day of therapy, the attainment of a target of 98%*f*T_>MIC_ was assessed. Total concentrations were evaluated due to the low (≈2%) protein binding of meropenem [26]. Furthermore, to assess target attainment based on a single observed minimum meropenem concentration and to limit toxicities arising from high minimum meropenem concentrations, an additional target was introduced: the target range for minimum plasma concentrations was defined to be 1–5xMIC [27].

### 2.4. Development of the Dosing Decision Tool

#### 2.4.1. Selection and Evaluation of Dosing Regimens

Dosing regimens were preselected based on their feasibility to integrate into local clinical routine. To reduce the number of eligible dosing regimens emerging from the possible combinations of the four variables (loading dose, infusion dose, infusion duration, dosing interval), deterministic simulations were performed. Comparing the dosing regimens, those achieving higher predicted minimum meropenem concentrations were further considered. The remaining dosing regimens (Table 1) were evaluated for probability of target attainment (PTA); for each dosing regimen and patient, the meropenem concentration time profile was predicted 1000 times (Monte Carlo simulations), and the probability to attain the PK/PD target was calculated for each individual MIC value). PTA was computed for treatment days 1 and 2 across target concentrations values ranging from 1 to 32 mg/L and creatinine clearance values were estimated according to Cockcroft and Gault (CLCRCG) [28] ranging from 10 to 300 mL/min (10–150 mL/min in steps of 10 mL/min, above in steps of 50 mL/min). PK model parameter uncertainty was incorporated by repeating each Monte Carlo simulation and the respective PTA analysis 1000 times using the PK parameter sets obtained from a non-parametric bootstrap. A dosing regimen leading to a PTA ≥ 90% for the median of the 1000 computed PTA values was considered adequate [29]. All dosing regimens reaching a PTA ≥ 90% were further ranked according to higher probability of minimum concentrations being in the defined target range (1–5xMIC) and subsequently according to lower total daily dose. Thus, for each CRCLCG group and MIC value, a single dosing recommendation was derived.

#### 2.4.2. Integration of Locally Available Pathogen Information

As a high number of antibiotic therapies are initiated prior to pathogen detection, dosing recommendations accounting for this situation were developed. Based on the PTA results and the pathogen-independent MIC distribution observed at Charité-Universitaetsmedizin Berlin in the previous year, the local pathogen-independent mean fraction of response (LPIFR) was introduced as metric for each dosing regimen: To determine the LPIFR for a dosing regimen ($LPIFR_{DR}$), first, the PTA for each investigated MIC level ($PTA_{MIC,DR}$) was multiplied by the relative MIC frequency ($\frac{n_{MIC}}{N_{MIC,total}}$; $n_{MIC}$ = number of MIC values observed at a MIC level, $N_{MIC,total}$ = total number of observed MIC values) at this level in the distribution of MIC values in patients treated at Charité-Universitaetsmedizin Berlin. Next, the resulting MIC-frequency weighted PTA values were summarised per dosing regimen (Equation (1)):

(1) $$LPIFR_{DR}=\sum_{MIC}\left({PTA}_{MIC,DR}\times \frac{n_{MIC}}{N_{MIC,total}}\right).$$

An LPIFR of ≥90% was considered adequate. Within each CRCLCG group, dosing regimens with a LPIFR ≥ 90% were selected and ranked by lower total daily dose.

### 2.5. Retrospective Evaluation of the Dosing Decision Tools Using Real Patient Data

Prior to implementation into clinical practice, the developed dosing decision tool was evaluated using the observed local patient population. The total daily dose of the dosing regimens recommended by the dosing decision tool for the local study population was compared to the total daily dose of the actual administered dosing regimens. For this purpose, the dataset of the local study population was stratified based on target attainment (above, below, and in the defined target range of 1–5xMIC) and the administered and recommended daily dose were compared.

## 3. Results

### 3.1. Pharmacokinetic Model Selection, Reduction, and Evaluation

A PK model developed by Ehmann et al. was selected for evaluation based on the high similarity in patient characteristics between the population used for model development and the local study population (Table 2 and Supplementary Material S1, Figure S1) [30]. The two-compartment model included a piecewise linear relation between CLCRCG and clearance (CL), a power relation between body weight and the central volume of distribution (V1), and a linear relation between serum albumin concentration and the peripheral volume of distribution (V2). Of these three covariates, Ehmann et al. demonstrated that only CLCRCG had a clinically relevant impact on PTA [30]. Therefore, for the development of the dosing tool, CLCRCG was kept as the only covariate in the model.

This new reduced PK model was a two-compartment model with first-order elimination, interindividual variability on CL, V1 and V2, inter-occasion variability on CL, and a combined proportional and additive residual variability model (Supplementary Material S1, Figure S2). For this new reduced model, the PK parameters were re-estimated using the original dataset of the full model [30]. CL was shown to linearly increase with increasing CLCRCG up to an inflection point of 154 mL/min. An extensive internal model evaluation of the reduced model demonstrated high parameter accuracy and precision, robustness and predictive performance and, thus, applicability of the PK model to the new population (Supplementary Material S1, Text and Figure S3).

**Figure 1:**
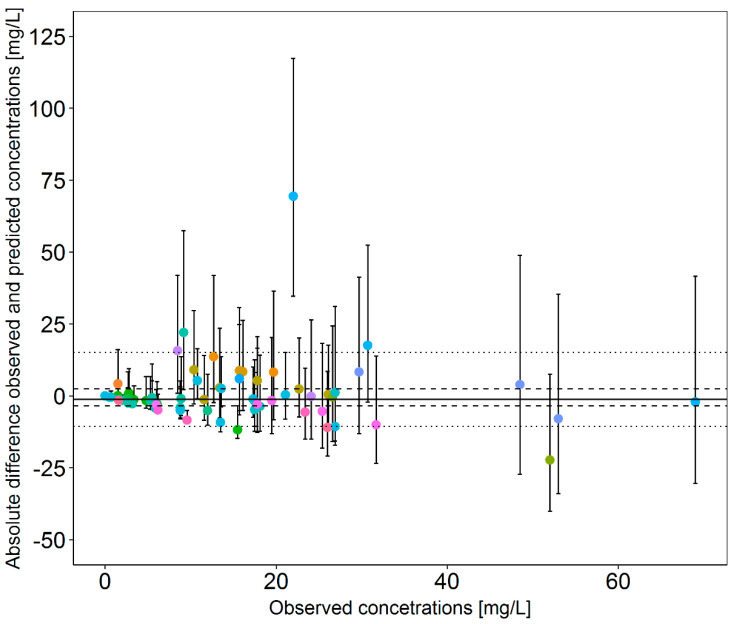
Absolute prediction error (mg/L) plotted against observed meropenem concentrations (n = 66) when predicting concentration based on the reduced pharmacokinetic model for the data in the subset. Points: median prediction error per sample. Colours: individual patients (i = 34). Error bar: 90% prediction interval of prediction error per sample. Solid horizontal line: median prediction error. Dashed line: 50% prediction interval of median prediction error. Dotted line: 90% prediction interval of median prediction error.

In Figure 1, prediction errors are plotted against observed meropenem concentrations in the 34 ICU patients with full dosing history. The median prediction error across all observations was −1.2 mg/L, indicating a slight bias towards underprediction. The 50% prediction error interval ranging from −3.5 to +2.5 mg/L indicated acceptable precision for the ICU patient population the model was applied to with a single outlier (Figure 1). Other samples of the same patient showed acceptable prediction errors, and therefore, this sample was excluded from the subsequent NPDE analysis. While the overall NPDE distribution did not significantly differ from the standard normal distribution (global adjusted *p*-value: 0.0976), the Wilcoxon signed-rank test revealed a significant (*p*-value 0.0325) deviation from a mean of 0 and therefore confirmed a small bias (NPDE mean: 0.296; detailed results: Supplementary Material S1, Tables S2 and S3; Figure S4).

### 3.2. Development of the Dosing Decision Tool

#### 3.2.1. Selection and Evaluation of Dosing Regimens

**Figure 2:**
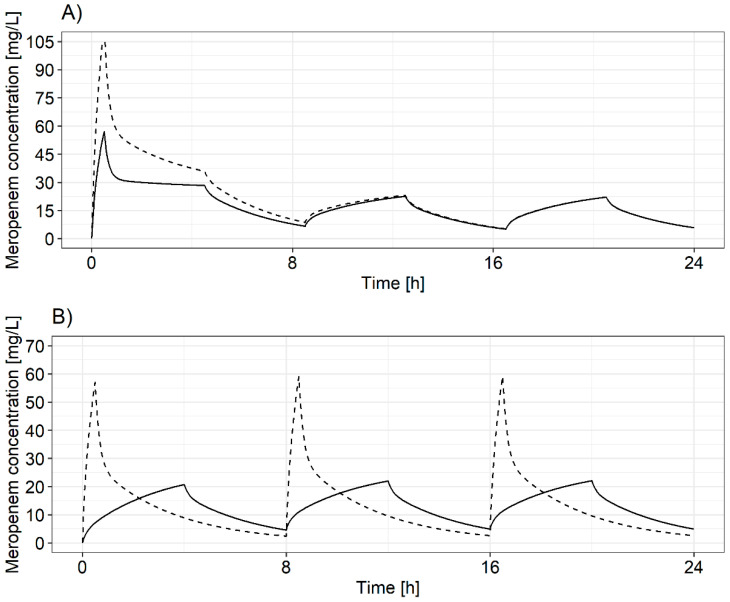
Predicted meropenem concentration–time profiles based on deterministic simulations using the reduced population pharmacokinetic model for a patient with creatinine clearance of 80.8 mL/min. (**A**) After either a 1000 (solid line) or a 2000 mg (dashed line) loading dose followed by prolonged (4 h) 1000 mg meropenem infusions with a dosing interval of 8 h. (**B**) After either a short-term (0.5 h; dashed line) or a prolonged (4 h; solid line) 1000 mg meropenem infusion administered every 8 h.

Deterministic simulations demonstrated that 2000 mg loading doses provided little further benefit over 1000 mg loading doses, the latter being sufficient to reach minimum meropenem concentrations above minimum meropenem concentrations in steady state (Figure 2A). Furthermore, short-term (0.5 h) infusions were inferior to prolonged infusions (4 h) with short-term infusions generally having higher maximum and lower minimum concentrations for the same daily dose (Figure 2B). Consequently, dosing regimens with a 2000 mg loading dose and short-term infusions were not further considered for PTA analysis. PTA analysis of the 15 remaining dosing regimens (Table 1) showed that for prolonged (4 h) infusions, four-times-daily dosing (i.e., a 6 h dosing interval) reached higher PTA values with lower total daily doses than three-times-daily dosing (Table 3). Furthermore, four-times-daily dosing of prolonged infusions (4 h) reached higher PTA values than continuous infusions with the same total daily dose, which was due to the higher targets for continuous infusions (Table 3). For pathogens with MIC ≥ 8 mg/L in patients with augmented renal clearance (≥150 mL/min), none of the investigated dosing regimens reached a PTA ≥ 90%.

**Figure 3:**
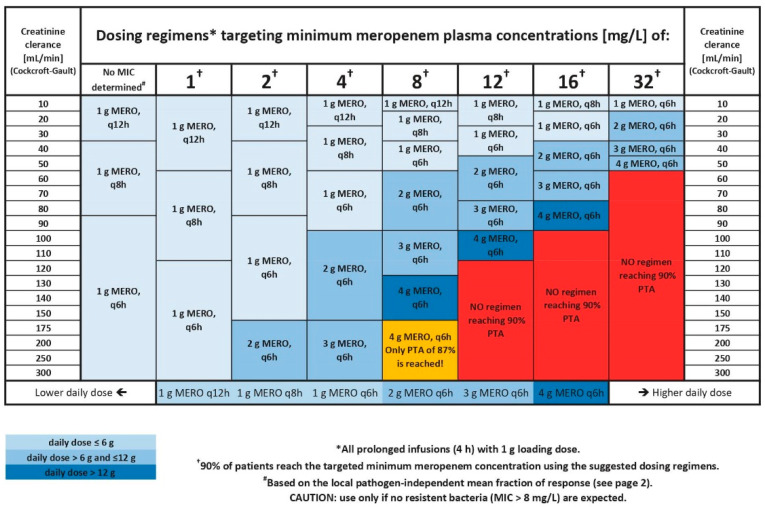
Front page of the developed dosing decision tool for initial meropenem dosing in intensive care patients. Dosing recommendations are stratified for creatinine clearance according to Cockroft and Gault and target (minimal meropenem concentration or local pathogen-independent mean fraction of response (LPIFR), see text) MERO: meropenem; q6h: every 6 h dosing; q8h; every 8 h dosing; q12h: every 12 h dosing; PTA: probability of target attainment; MIC: minimal inhibitory concentration.

Finally, dosing recommendations stratified by patient’s CLCRCG and determined MIC were summarised in a single concise table (Figure 3).

#### 3.2.2. Integration of Locally Available Pathogen Information

If the pathogen and its MIC value are not known at the time of dosing selection, two options are implemented in the dosing tool: an empirical dosing regimen based on non-species related EUCAST breakpoints for meropenem or a dosing regimen based on the LPIFR metric and pathogen-independent MIC distribution data from ICUs at Charité-Universitaetsmedizin Berlin. A short summary of both options was added on the backside of the dosing decision table (Supplementary Material S2). Compared to targeting the pathogen independent ‘susceptible/susceptible at increased exposure’ (2 mg/L) or the ‘susceptible at increased exposure/resistant’ (8 mg/L) EUCAST breakpoints, the LPIFR substantially reduced the drug exposure in patients while still assuring a desired percentage of 90% of patients being above the PK/PD target of 98%T_>MIC_. Based on the LPIFR, the daily dose for a patient with a creatinine clearance of 120 mL/min was 4000 mg, whereas there was a three-fold higher dose of 12,000 mg when targeting the EUCAST susceptible at increased exposure/resistant breakpoint (8 mg/L).

### 3.3. Retrospective Evaluation of the Dosing Decision Tools Using Real Patient Data

**Figure 4:**
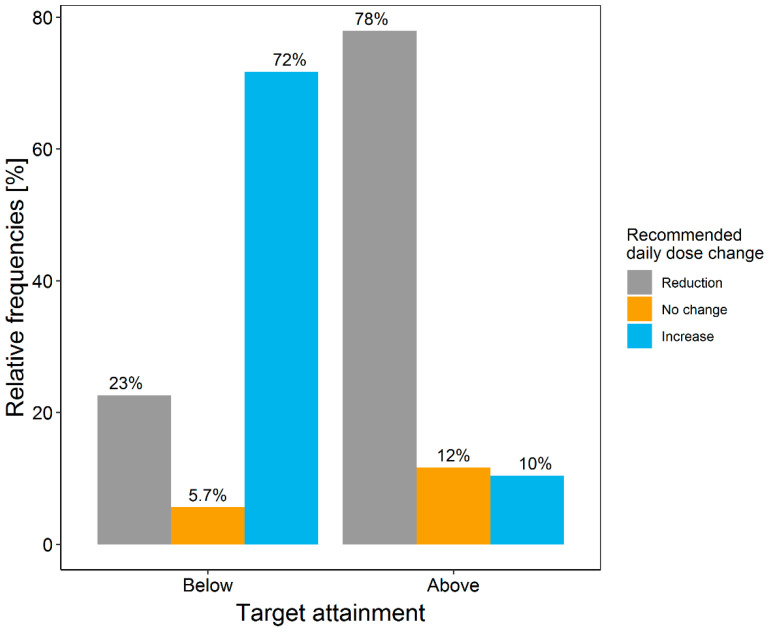
Frequency of daily meropenem dose adjustments by comparing the recommended daily dose to the actual administered daily dose at Charité-Universitaetsmedizin Berlin, stratified by non-attainment of the target range of the administered dosing regimen. Of 306 samples, 46 were below and 160 were above the target range.

Of the 306 meropenem samples of the local study population, 46 (15.0%) were found to be below and 160 (52.3%) were found to be above the defined target range. The retrospective application of the developed tool recommended a change in dosing for the majority (77%) of patients with concentrations observed outside the target range (Figure 4). For 72% of the patients with minimum meropenem concentration below the target range, the developed dosing tool recommended an increased daily dose, while for 78% of the patients with samples above the target range, a lower daily dose was recommended.

## 4. Discussion

**Figure 5:**
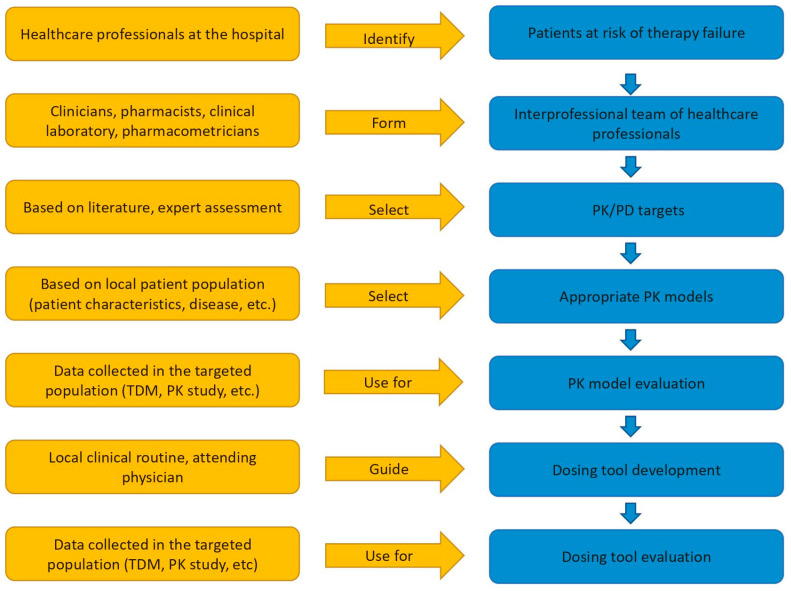
Steps (blue) considered in the generalised workflow development of a tabular precision dosing tool for initial therapy together with recommendations (yellow) to support each step.

A concise dosing decision tool for initial meropenem dosing incorporating local susceptibility data was developed for the specific local needs. Additionally, a generalised workflow that can be used as blueprint for the development of such a dosing decision tool at the point-of-care was derived based on our experiences (Figure 5). After the identification of the elevated risk of inadequate antibiotic drug exposure in ICU patients, a local collaboration was established to assess and, if needed, improve antibiotic dosing. The close interprofessional collaboration between the antimicrobial stewardship (AMS) team, infectious disease specialists, critical care specialists, pharmacists, the clinical laboratory and pharmacometricians proved to be a vital part of the development process and should enable best adaptation to the local clinical routine. Bi-weekly meetings of the study team enabled continuous discussions, feedback, and adjustments throughout each step of the course of action.

As a mandatory prerequisite, the external PK model evaluation assured good predictive performance between the developed tool and the local patient population. The slight bias of the PK model to underpredict observed meropenem concentrations led to slightly lower PTA values for each dosing regimen and can be considered as an additional safety margin. The first evaluation of the dosing decision tool using retrospectively collected data suggests a substantial potential to improve target attainment. For 72% of the patients with concentrations below the target, a dose increase was recommended, and for 78% of the patients with concentrations above the target, a dose reduction was recommended. The suggested reduction of daily dose for 23% of samples below the target is due to the recommendation of four-times-daily dosing instead of the three-times-daily dosing being administered: This more frequent administration of meropenem achieved higher PTA values despite reduced daily doses (Table 3). At the same time, the suggested increase in daily dose for 10% of the sample above the target range is mostly likely due to the selected PK model: The safety margin included in the PK model leads to more conservative, higher dosing recommendations to guarantee effective drug exposure. Additionally, in both cases, the high PK variability observed in critically ill patients renders a perfect recommendation for all patients untenable. To conclude, the retrospective evaluation highlighted the potential of the tool to improve meropenem therapy in critically ill patients. As next step, a prospective clinical trial should investigate the impact of the dosing decision tool on target attainment. The approach and workflow presented can improve acceptance and therefore the implementation of model-informed dosing decision tools at the point-of-care.

All recommended dosing regimens included a 1000 mg loading dose to ensure an immediate achievement of sufficiently high meropenem concentrations at the start of antibiotic therapy. Regardless of the loading dose used (1000 mg vs. 2000 mg), the minimum meropenem concentration after the first maintenance dose was higher than or equal to minimum concentrations in steady state. Therefore, a 2000 mg loading dose showed no additional benefit compared to a 1000 mg loading dose and was considered to be an unnecessary higher drug exposure for patients and thus not retained in the dosing tool. Due to the different PK/PD targets for prolonged and continuous infusions, prolonged infusions provided higher target attainment for the same daily dose and as a consequence represent all integrated dosing regimen. In order to keep an explicit and clear structure in the dosing decision tool, only one dosing regimen was incorporated for each individual CLCRCG and MIC value. Overall, only six different initial dosing regimens (Table 2) were included in the dosing decision tool. This simplicity aims to achieve an initial dose individualisation to optimise dosing in ICU patients while maintaining a level of standardisation and avoiding complication of the ICU ward process. One further important note on the integrated dosing regimen: The dosing regimens selected and integrated into the tool were selected based on their ability to reach a predefined PK target. As a consequence, high daily doses (up to 16 g) are included in the tool for high target concentrations. However, for such high targets, a change in antibiotic should be considered.

A further feature in the dosing decision tool is the use of local, hospital-specific and pathogen-independent MIC values. The LPIFR metric facilitates dosing based on not only patient characteristics but additionally on local bacterial susceptibility conditions in the hospital. While we foresee that this approach is a valuable opportunity to reduce unnecessary high meropenem dosing, we also strongly advise to apply it with caution: As local MIC distributions can vary over time, these need to be monitored and dosing suggestions based on LPIFR need to be updated regularly. Furthermore, in the rare event of a pathogen with high MIC values (>8–32 mg/L), the risk of target non-attainment could be underestimated until the MIC is determined. Therefore, it is vital to communicate those limitations to the decision-making team and encourage a dosing increase or change of antibiotic if higher MIC values are expected. In general, the dosing regimen recommendations based on the LPIFR metric should only be used as long as there is no further information about the pathogen (e.g., MIC) available. Furthermore, patients receiving renal replacement therapy (RRT), obese patients, and paediatric patients were not included in the PK model development and evaluation. Consequently, the derived dosing recommendations do not apply to those patient populations but only to adult critically ill patients.

For critically ill patients receiving antibiotics, drug measurements linked with Bayesian dosing software have been recommended for dose individualisation [3]. To date, this very promising approach could not always be implemented into clinical practice. Individualised meropenem therapy guided by concentration measurements is still only available in very few hospitals [10]. As an alternative, tabular dosing decision tools based on PTA analysis of an evaluated PK model can be used for initial dosing. Furthermore, these tabular dosing decision tools can provide dosing recommendations prior to the first drug measurement to improve dosing in this especially crucial time window of antibiotic drug therapy [31].

To optimise meropenem dosing, several complementary model-based tools or algorithms exist: the MeroRisk calculator supports the identification of critically ill patients at risk of suboptimal exposure and different model-based algorithms or nomograms provide dosing suggestions [30,32,33]. Unfortunately, none of the tools matched our local conditions and objectives: The dosing regimens frequently used at Charité-Universitaetsmedizin Berlin were either not included in the available tools or were part of a multitude of dosing regimen recommendations complicating the daily use of the tool. Furthermore, the risk of reaching toxic minimum concentrations was not considered in those other tools, and an evaluation of the underlying PK models would have been necessary. Most likely, this situation is similar for a wide range of drugs, model-based tools, and hospitals. Even when model-based tools or PK models are available for a specific drug in a specific patient population, the selected target or the clinical setting might hinder implementation and use. In those situations, local initiatives are needed to develop a dosing decision tool fit for the situation on site. As presented in our example, routine drug measurements can be used to evaluate published PK models instead of conducting expensive clinical trials to develop new PK models as the basis for new tools. Furthermore, by employing the LPIFR, antibiotic dosing prior to pathogen detection can be adapted to local susceptibility patterns. Even though tools developed to fit local conditions might be more difficult to transfer to other institutions, we believe the advantages of local initiatives clearly outweigh this drawback. The approach with the generalised workflow (Figure 5) may serve as a blueprint to a wide range of hospitals, patient populations, and drugs to develop a sophisticated dosing decision tool providing optimised initial dosing adapted for local conditions and objectives.

## Figures and Tables

**Table 1:** Meropenem dosing regimens investigated in probability of target attainment analysis for potential inclusion in the dosing decision tool.

Dosing Regimen	Dose Per Infusion [mg]	Infusion Duration [h]	Dosing Interval [h]	Total Daily Dose [mg]
1	1000	4	6	4000
2	1000	4	8	3000
3	1000	4	12	2000
4	2000	4	6	8000
5	2000	4	8	6000
6	2000	4	12	4000
7	3000	4	6	12,000
8	3000	4	8	9000
9	3000	4	12	6000
10	4000	4	6	16,000
11	4000	4	8	12,000
12	4000	4	12	8000
13	4000	24	24	4000
14	6000	24	24	6000
15	8000	24	24	8000

All dosing regimens were administered in combination with a 1000 mg meropenem loading dose; *Grey*: Dosing regimen selected for the developed dosing tool.

**Table 2:** Overview of patient characteristics.

Patient Characteristic	Charité Universitätsmedizin-Berlin	Ehmann et al.
Categorical	n (%)	n (%)
No. of patients	81	42
No. of meropenem samples	306	1376
Male	55 (67.9)	27 (56.3)
No. of extracorporeal membrane oxygenation	8 (9.88)	6 (12.5)
Continuous (unit)	Median (5th–95th percentile)	Median (5th–95th percentile)
Age (years)	64.0 (40.0–81.0)	55.5 (32.0–69.9)
Weight (kg)	75.0 (48.0–116)	70.5 (47.4–121)
Creatinine clearance ^#^ (mL/min)	74.4 (24.7–253)	80.8 (24.8–191)
Serum albumin concentration (g/dL)	2.68 (2.00–3.60)	2.80 (2.20–3.56)

^#^ Calculated using Cockcroft–Gault formula [28]. Creatinine clearance and serum albumin concentration determined on sample level, all other characteristics determined on patient level.

**Table 3:** Probability of target attainment for different dosing regimens administered to a patient with a creatinine clearance according to Cockroft–Gault of 120 mL/min and infected by a pathogen with a minimum inhibitory concentration of 4 mg/L for meropenem.

Total Daily Dose [mg]	Probability of Target Attainment, %		
	PI, 6 h Interval	PI, 8 h Interval	CI, 24 h Interval
4000	75.0	-	16.2
6000	-	58.0	67.7
8000	97.0	-	67.6
9000	-	76.8	-
12,000	99.4	-	-

Abbreviations: PI: Prolonged (4 h) infusion, CI: Continuous infusion.

## Data Availability

The data presented in the study are available at reasonable request from the corresponding author.

## Supplementary Materials

The following are available online at https://www.mdpi.com/article/10.3390/pharmaceutics13122128/s1, Supplementary Material S1: Pharmacokinetic model selection, reduction and evaluation, Supplementary Material S2: Dosing decision tool.
