# Supplementary material for: Development of a Model-Informed Dosing Tool to Optimise Initial Antibiotic Dosing—A Translational Example for Intensive Care Units
Source: Pharmaceutics. 2021 Dec 10;13(12):2128. doi: 10.3390/pharmaceutics13122128 (PMC8708464; doi:10.3390/pharmaceutics13122128)
Supplement: Supplementary file 1 [file pharmaceutics-13-02128-s001.zip › pharmaceutics-1428206-supplementary material/pharmaceutics-1428206 - supplementary material S1_corrected.pdf]

pharmaceutics

Supplementary Materials S1: Development of a Model-Informed Dosing Tool to Optimise Initial Antibiotic Dosing—A Translational Example for Intensive Care Units

Ferdinand Anton Weinelt, Miriam Songa Stegemann, Anja Theloe, Frieder Pfäfflin, Stephan Achterberg, Lisa Schmitt, Wilhelm Huisinga, Robin Michelet, Stefanie Hennig and Charlotte Kloft

Model Selection, Reduction and Evaluation

Methods

Modeling activities were performed in NONMEM 7.4.3 (ICON Development Solutions, Ellicott City, MD, USA) and PsN version 4.7.0 [20]. First-order conditional estimation with interaction (FOCE+I) was utilised for parameter estimation; data visualisations were performed in R/RStudio (v. 3.5.0/v. 1.1.447, RStudio, 250 Northern Ave, Boston, MA 02210). The Ehmann et al. model [30] was selected for further evaluation based on the high similarity of patient characteristics (disease scores, body weight, height, creatinine concentration, albumin concentration) between the local study population and the underlying population (Figure S1). The reduced PK model for meropenem was evaluated using standard goodness-of-fit plots, e.g. population and individual prediction versus observations, conditional weighted residuals versus time or versus population predictions. To assess the predictive performance, a visual predictive check (VPC, n = 1000 simulations) was performed. The accuracy and precision of the PK parameter estimates, as well as model robustness (evaluating the convergence rate) were assessed performing non-parametric bootstraps sampling form the original dataset (n = 1000 replicate datasets).

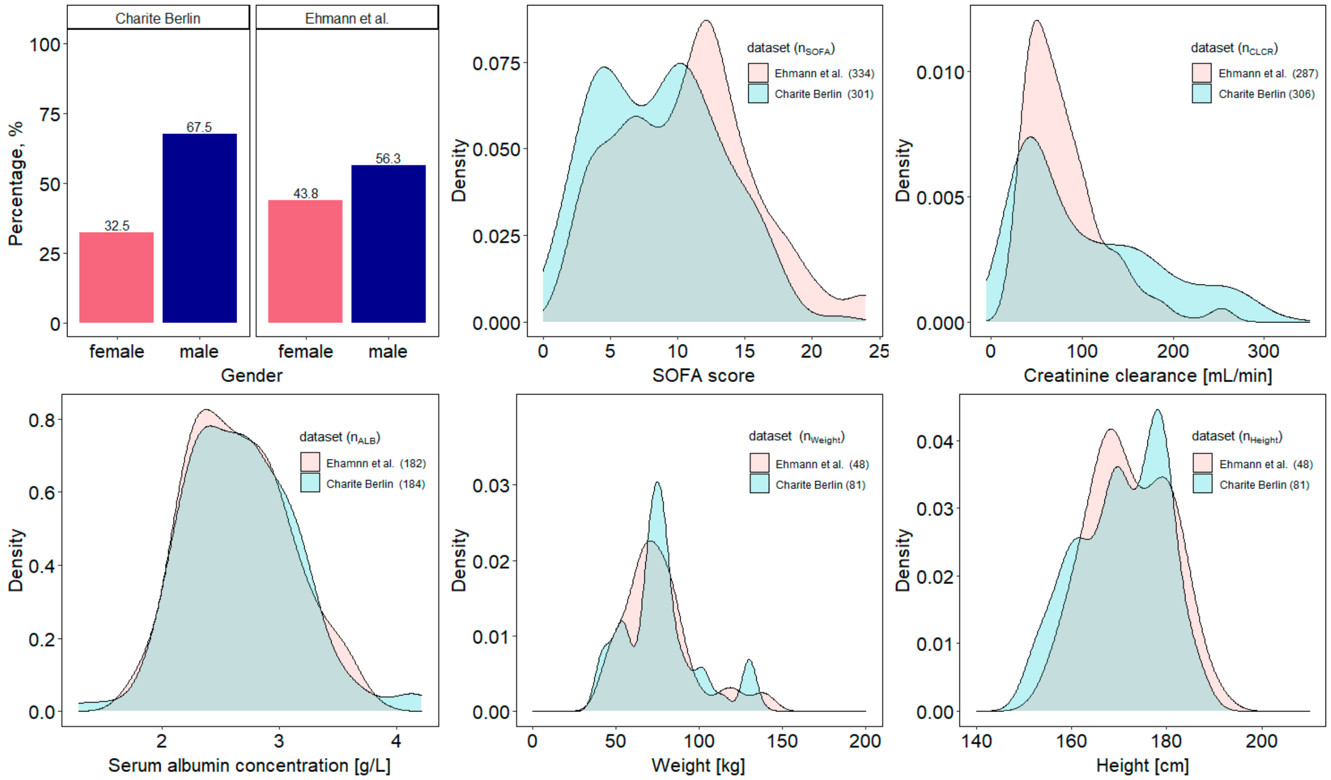

Figure S1. Comparison of relevant patient characteristics between the local study population and the population investigated by Ehmann et al. [30]
Abbreviations: *SOFA*: Sequential Organ Failure Assessment; *CLCR*: Creatinine clearance; *ALB*: serum albumin concentration.

Reduced population pharmacokinetic model

A sketch of the developed population PK model is displayed in Figure S2.

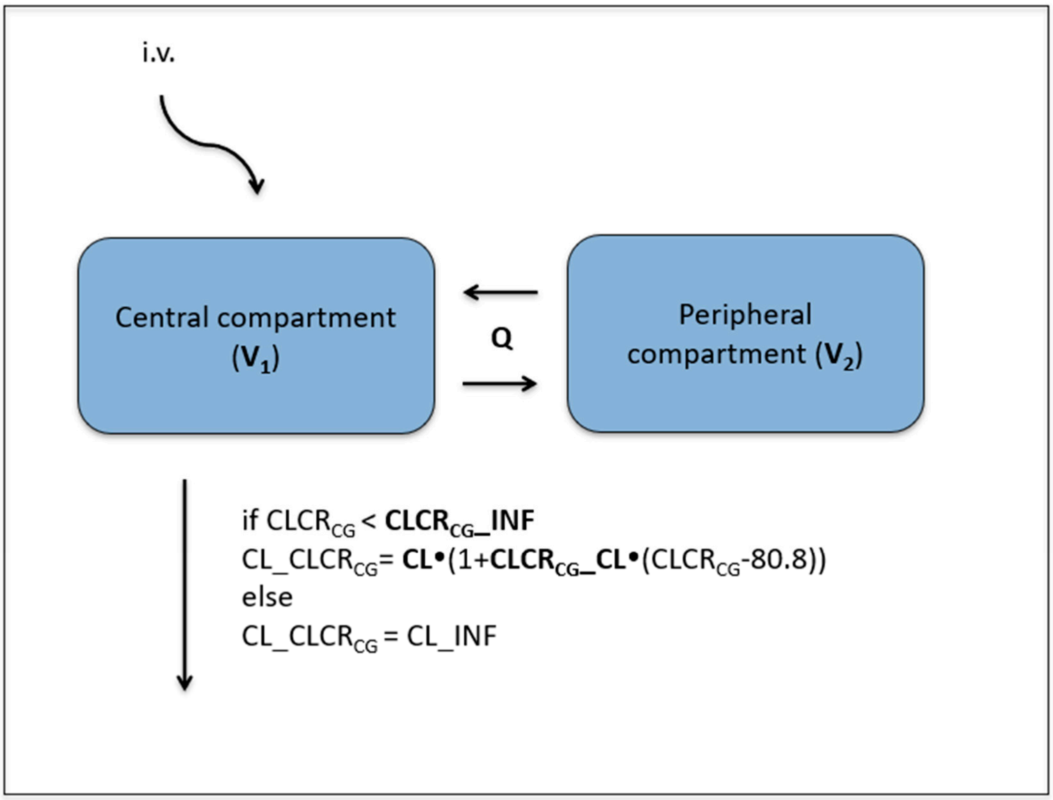

Figure S2. Model sketch of the final population pharmacokinetic model. **Bold:** Estimated fixed-effects parameters. Abbreviations: *CL*: Typical clearance for median $CLCR_{GC}$; *CL_CLCR_{CG}*: typical clearance for a given $CLCR_{CG}$ value; $CLCR_{CG}$: Creatinine clearance estimated according to Cockcroft and Gault [28]; *$CLCR_{CG}\_CL$*: $CLCR_{CG}$ effect on CL; *$CLCR_{CG}\_INF$*: $CLCR_{CG}$ value serving as inflection point; *CL_INF*: CL at $CLCR_{CG}$_INF (= maximum clearance) = $CL \cdot (1+CLCR_{CG}\_CL \cdot (CLCR_{CG}\_INF-80.8))$; *Q*: Typical intercompartmental clearance; *V_1*: Typical central volume of distribution; *V_2*: Typical peripheral volume of distribution

Results

Standard goodness-of-fit plots indicated adequate model predictions; VPC revealed good predictive performance both for the typical trend and variability of the meropenem concentration-time profiles (see Figure S3). The non-parametric bootstrap confirmed model robustness (convergence rate = 90.4%) and narrow 95% confidence intervals of the bootstrap parameter estimates, which overall were very similar to the parameter estimates of the reduced model (Table S1).

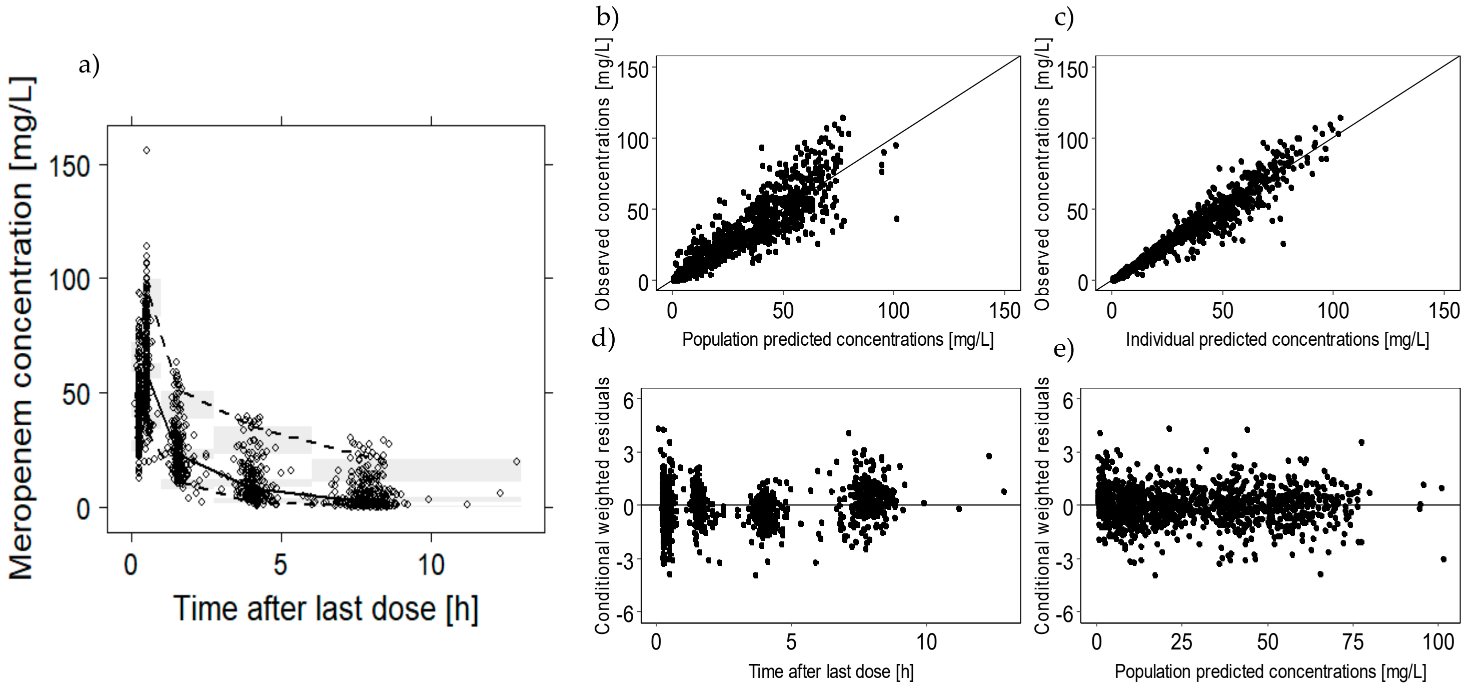

Figure S3. Visual predictive check (**a**), n = 1000 simulations) and goodness-of-fit plots (**b**–**e**)) for the final reduced population pharmacokinetic model of meropenem. (**a**): Circles: Observations, Lines: 5th, 95th percentile (dashed), 50th percentile (solid) of the observed data. Shaded areas: 95% confidence interval around 5th, 50th and 95th percentile of simulated data. (**b**,**c**) : Lines: Line of unity. d, e: Horizontal lines: Reference lines at y = 0.

Table S1. Parameter estimates for the reduced pharmacokinetic model of meropenem.

Parameter [unit]	Final Model Estimate (RSE; 95% CI[a])	Bootstrap[b] Median (95% CI)
	Fixed-effects parameters	
CL[c] [L/h]	9.25 (4.5; 8.43–10.1)	9.29 (8.39–10.2)
V_1 [L]	8.22 (11.8; 6.32–10.1)	8.14 (6.27–11.8)
Q [L/h]	28.3 (15.8; 19.5–37.1)	28.7 (11.4–37.6)
V_2 [L]	16.3 (7.40; 13.9–18.6)	16.2 (12.3–18.7)
$CLCR_{CG}$_CL	0.00984 (9.10; 0.00808–0.0116)	0.00993 (0.00725–0.0113)
$CLCR_{CG}$_INF [mL/min]	154 (7.20; 132–176)	154 (113–173)
	Interindividual variability (IIV) parameters	
CL, %CV	27.1 (19.5; 16.7–37.5)	26.6 (17.3–38.0)
V_1, %CV	41.5 (12.1; 31.7–51.3)	40.7 (27.1–53.0)
V_2, %CV	20.2 (15.0; 14.3–26.1)	20.1 (12.1–28.9)
	Interoccasion variability (IOV) parameters[d]	
CL, %CV	12.7 (12.4; 9.61–15.8)	12.4 (9.27–15.4)
	Residual variability (RUV) parameters	
Proportional, %CV σP_{tot}	16.5 (6.30; 14.5–18.5)	16.4 (14.2–18.6)
Additive, SD [mg/L]	0.251 (26.1; 0.122–0.379)	0.243 (0.110–0.343)

[a]Computed as: parameter estimate ± 1.96*SE

[b]Non-parametric bootstrap (n = 1000): convergence rate of 90.4%.

[c]CL for median $CLCR_{CG}$ of non-CRRT patients on first study day (80.8 mL/min).

[d]Occasion was defined as monitored meropenem infusion.

Abbreviations: *CI*: Confidence interval; *CL*: Clearance; *$CLCR_{CG}$*: Creatinine clearance estimated according to Cockcroft and Gault [28]; *$CLCR_{CG}$_CL*: $CLCR_{CG}$ effect on CL; *$CLCR_{CG}$_INF*: $CLCR_{CG}$ value serving as inflection point. *CRRT*: Continuous renal replacement therapy, *%CV*: Coefficient of variation (calculated as exemplified for IIV: IIV, %CV=$\sqrt{e^{IIV^2}-1}\cdot 100$); *Q*: Intercompartmental clearance; *RSE*: Relative standard error (RSE of random effects parameters reported on approximated standard deviation scale); *SD*: Standard deviation; *SE*: Standard error; *V_1*: Central volume of distribution; *V_2*: Peripheral volume of distribution.

Normalised prediction distribution errors

Table S2. Distribution of normalised prediction distribution errors (NPDE).

Distribution of NPDEs	Value
Mean	0.2958 (se = 0.14)
Variance	1.19 (se = 0.21)
Skewness	−0.0491
kurtosis	−0.4235

se: standard error

Table S3. Statistical tests to assess normality assumption.

Statistical Test	p-value
Wilcoxon signed-rank test	0.0325
Fisher ratio test	0.283
Shapiro-Wilks test	0.784
**Global adjusted p-value**	**0.0976**

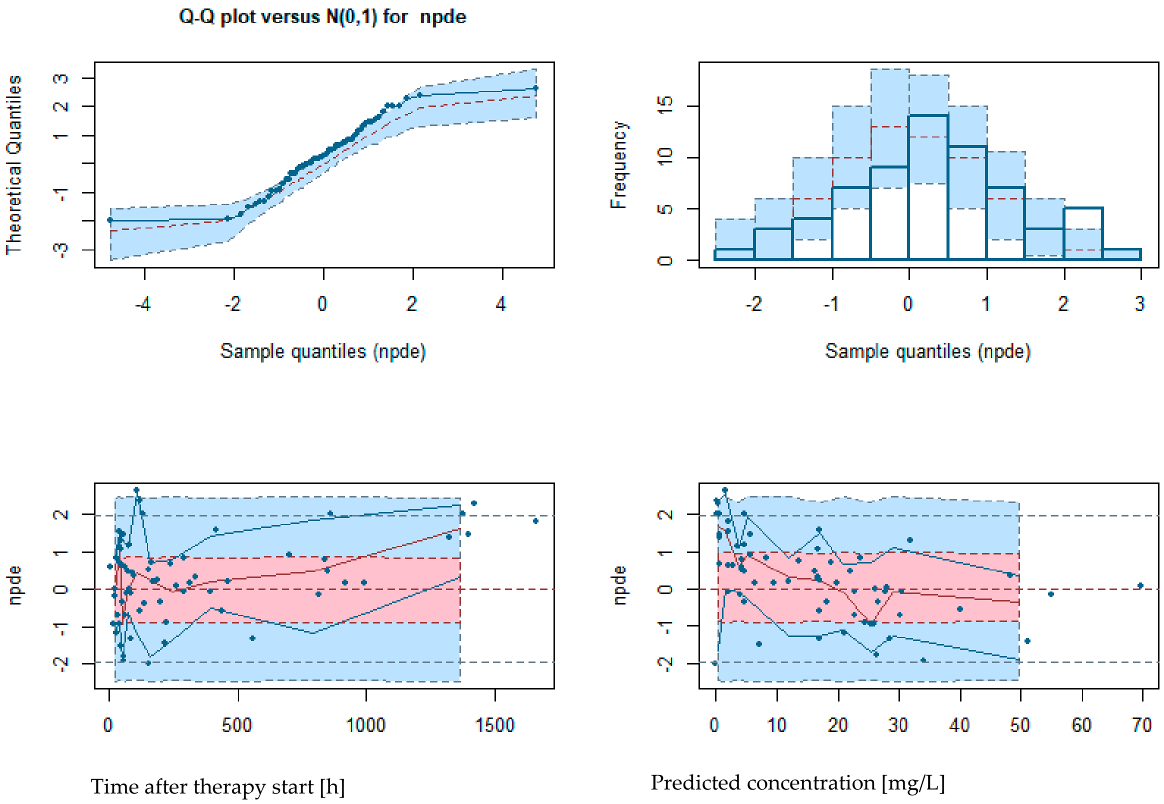

Figure S4. Graphical output of the normalised prediction distribution error (NPDE) analysis. Upper left: Quantile-quantile plot of NPDE versus the expected standard normal distribution. Upper right: Histogram of NPDE with the density of the standard normal distribution overlaid. Lower left: Scatterplot of NPDE versus time after therapy start. Lower right: Scatterplot of NPDE versus predicted concentration. Pink area*:* Prediction interval for the median, Blue area: 95% prediction interval.

References:

21. Lindbom, L., Pihlgren, P. & Jonsson, N. PsN-Toolkit - A collection of computer intensive statistical methods for non-linear mixed effect modeling using NONMEM. *Comput. Methods Programs Biomed.* **2005**, *79*, 241–257.
28. Cockcroft, D. W. & Gault, M. H. Prediction of Creatinine Clearance from Serum Creatinine. *Nephron* **1976**, *16*, 31–41.
30. Ehmann, L.; Zoller, M.; Minichmayr, I.K.; Scharf, C.; Huisinga, W.; Zander, J.; Kloft, C. Development of a dosing algorithm for meropenem in critically ill patients based on a population pharmacokinetic/pharmacodynamic analysis. *Int. J. Antimicrob. Agents* **2019**, *54*, 309–317.
